# Phosphorylation of serine 349 of p62 in Alzheimer’s disease brain

**DOI:** 10.1186/2051-5960-2-50

**Published:** 2014-05-03

**Authors:** Kunikazu Tanji, Yasuo Miki, Taku Ozaki, Atsushi Maruyama, Hidemi Yoshida, Junsei Mimura, Tomoh Matsumiya, Fumiaki Mori, Tadaatsu Imaizumi, Ken Itoh, Akiyoshi Kakita, Hitoshi Takahashi, Koichi Wakabayashi

**Affiliations:** Department of Neuropathology, Institute of Brain Science, Hirosaki University Graduate School of Medicine, 5 Zaifu-cho, Hirosaki, 036-8562 Japan; Department of Ophthalmology, Hirosaki University Graduate School of Medicine, Hirosaki, Japan; Department of Stress Response Science, Hirosaki University Graduate School of Medicine, Hirosaki, Japan; Department of Vascular Biology, Hirosaki University Graduate School of Medicine, Hirosaki, Japan; Department of Pathological Neuroscience, Center for Bioresource-based Research, Brain Research Institute, University of Niigata, Niigata, Japan; Department of Pathology, Brain Research Institute, University of Niigata, Niigata, Japan

**Keywords:** Alzheimer’s disease, Autophagy, Cytoplasmic inclusion, Keap1, Oxidative stress, p62/SQSTM1/sequestosome 1, Phosphorylation, Proteasome

## Abstract

**Background:**

Extensive research on p62 has established its role in oxidative stress, protein degradation and in several diseases such as Paget’s disease of the bone, frontotemporal lobar degeneration and amyotrophic lateral sclerosis. Importantly, previous studies showed that p62 binds directly to Keap1, which is a ubiquitin E3 ligase responsible for degrading Nrf2. Indeed, colocalisation of p62 and Keap1 occurs in tumorigenesis and neurodegeneration. A serine (S) residue in the Keap1-interacting region of p62 is phosphorylated in hepatocellular carcinoma, and this phosphorylation contributes to tumour growth through the higher affinity of p62 to Keap1. However, it remains largely unknown whether p62 is phosphorylated in the Keap1-interacting region under neurodegenerative conditions.

**Results:**

To answer this question, we generated an antibody against phosphorylated S349 (P-S349) of p62 and showed that S349 is phosphorylated following disruption of protein degradation. In particular, the ratio of P-S349 to total p62 levels was significantly increased in the brains with Alzheimer’s disease (AD) compared with controls. We also compared the reactivity of the P-S349 antibody with P-S403 of p62 and showed that these two phosphorylated sites on p62 cause different responses with proteasome inhibition and show distinct localisation patterns in AD brains. In addition to disruption of protein degradation systems, activation of oxidative stress can induce P-S349.

**Conclusion:**

These results support the hypothesis that disruption of protein degradation systems and sustained activation of the Keap1-Nrf2 system occur in the brains with AD.

**Electronic supplementary material:**

The online version of this article (doi:10.1186/2051-5960-2-50) contains supplementary material, which is available to authorized users.

## Introduction

Accumulation of misfolded or abnormally modified proteins is a major characteristic of many neurodegenerative diseases and is largely attributed to aging, oxidative stress, and genetic and environmental factors. Additionally, protein aggregates can occur in any situation causing intracellular disruption of the protein degradation system. Two major systems for protein degradation exist in mammals, the autophagy-lysosome system and the ubiquitin-proteasome system. Both systems cooperatively play an important role in intracellular protein degradation in the brain. Further studies using a brain-specific deletion of each system have shown that mice exhibit neurological deficits with age and that misfolded proteins are accumulated in neurons [1, 2].

p62/SQSTM1/sequestosome 1 (referred to as p62) is a multifunctional protein that is highly involved in protein degradation. p62 contains a ubiquitin-associated (UBA) domain at the C-terminus, thereby interacting with ubiquitinated and misfolded proteins [3, 4]. Additionally, p62 binds to one of the proteasomal subunits, regulatory particle 1 (Rpt1), through Phox and Bem1p (PB1) domains at the N-terminus [5]. In addition, p62 interacts with the autophagy-related gene (ATG) 8 family [6], which is essential to autophagosomal formation [7]. Because of its unique properties, it has been suggested that p62 functions as an adaptor protein to transport ubiquitinated and misfolded proteins for proteasomal and autophagic degradation. Importantly, because p62 itself is degraded by autophagy [8], increased levels of the p62 protein suggests that autophagic flux is impaired.

Recently, we assessed the level of p62 in the brains of patients with neurodegenerative dementia, Alzheimer’s disease (AD) and dementia with Lewy bodies (DLB), and showed that the level of p62 was significantly increased in the brains of patients with AD relative to controls [9, 10]. Furthermore, consistent with previous reports [11–13], several genes related to the stress response and detoxification were also increased in the brains with AD compared with controls. Interestingly, recent studies have shown that p62 binds directly to Keap1 [14–17], which functions as a stress sensor through regulation of NF-E2 related factor 2 (Nrf2) [18]. p62 is reported to be one of the Nrf2-target genes and was also identified as an antioxidant-responsive gene [15, 19]. These findings suggest a tight relationship between stress responses and protein degradation dysfunction.

In this study, we focused on the binding region of p62 with Keap1 (amino acids 344–356 of human p62). Notably, Hancock et al. and Ichimura et al. demonstrated that phosphorylation of serine 349 (S349) enhanced the binding affinity between Keap1 and p62 [20, 21]. However, it remains unclear whether this phosphorylation occurs in neurodegenerative conditions. Here, we generated an antibody specific to S349 of p62 and demonstrated that S349 was phosphorylated in the brains of patients with AD, with levels significantly higher in AD relative to controls. Further studies showed that S349 on p62 was phosphorylated upon disruption of the protein degradation systems and exposure to sustained oxidative stress.

## Materials and methods

### Primary antibodies

For generation of antibodies against phosphorylated p62 at S349, rabbits were immunised with a synthetic peptide based on residues 344–354 of human p62 with phosphorylated S349 (P-S349), conjugated at the amino terminus by an additional cysteine to keyhole limpet hemocyanine together with adjuvants. Antisera were purified by obtaining flow-through fractions from a column conjugated with p62 peptide. Phosphorylated p62 was characterised by ELISA by plating immunogen peptide in multiwell plates. To demonstrate specificity, the rabbit antisera against P-S349 were pre-absorbed with phosphorylated peptide. After centrifugation, the supernatant was filtered and used for experiments. For immunohistochemical studies, treatment with alkaline phosphatase (NEB, Beverly, MA, USA) was also performed before incubation with the primary antibody. An anti-p62 antibody specific to phosphorylation at S403 of human p62 was purchased from Millipore (Bedford, MA, USA) [22].

### Subjects

Tissue samples were obtained from the Department of Neuropathology, Institute of Brain Science, Hirosaki University Graduate School of Medicine, Hirosaki, and the Department of Pathology, Brain Research Institute, University of Niigata, Niigata, Japan. Written informed consent for autopsy, collection of samples and subsequent analysis was obtained from the next of kin of the deceased involved in this study. This study was approved by the Committee of Medical Ethics of Hirosaki University Graduate School of Medicine, Hirosaki, Japan. Brain tissues from patients with AD (n = 8) and normal controls (n = 8) were used (Table 1). The diagnoses had been confirmed by neuropathological examinations using immunohistochemistry for phosphorylated tau and amyloid β.

**Table 1:** **Summary of human subjects**

Case no.	Pathologic diagnosis	Age, y	Gender	PMI, h
1	AD	79	M	4
2	AD	86	F	4
3	AD	89	F	4
4	AD	81	F	4
5	AD	87	F	3
6	AD	87	F	3
7	AD	100	F	3
8	AD	104	F	3.5
9	control	71	M	1.5
10	control	76	M	3.2
11	control	82	F	4.5
12	control	71	M	1.5
13	control	75	M	1
14	control	64	M	3
15	control	62	M	9
16	control	82	M	2

AD, Alzheimer’s disease; PMI, postmortem interval.

### Cell cultures and treatment

SH-SY5Y human neuroblastoma (ECACC, Salisbury, Wiltshire, UK) and HeLa (JCRB, Osaka, Japan) cells were maintained in Dulbecco’s modified Eagle’s medium supplemented with 10% fetal calf serum and antibiotics. Cells were treated with epoxomicin (Calbiochem, San Diego, CA, USA), MG132 (Calbiochem), bortezomib (Cell Signaling Technology, Inc., Danvers, MA, USA), bafilomycin A (Wako, Osaka, Japan), and amyloid β peptide 1–40 or 1–42 (Aβ40 or Aβ42) (Peptide Institute Inc., Osaka, Japan). For activation of the Nrf2 system, diethyl maleate (DEM) was used at 100 μM. At the indicated times, the cultures were washed with phosphate-buffered saline (PBS) (pH 7.4), harvested and used as samples for further studies.

Human p62 cDNA was prepared as previously described [23]. p62 cDNA was subcloned into pcDNA3 (Invitrogen, Carlsbad, CA, USA) tagged with hemagglutinin (HA) or pEGFP-N1 (Invitrogen). Mutagenesis was performed according to the manufacturer’s instructions (Takara, Otsu, Japan), followed by sequencing to confirm the mutation site. Serine (S) was changed into glutamic acid (E) for a phosphorylation-mimetic mutant, or into alanine (A) for a phosphorylation-deficient mutant. Cells were transfected using Fugene 6 (Roche Molecular Biochemicals, Indianapolis, IN, USA) or Lipofectamine 2000 (Invitrogen). siRNAs were purchased from Dharmacon (Lafayette, CO, USA). The siRNAs (final concentration 20 nM) for p62 (5’-GCA TTG AAG TTG ATA TCG A-3’), Keap1 (M-012453-00-0005), and Nrf2 (M-003755-02-0005) were performed using Lipofectamine RNAi MAX (Invitrogen). After 24 h, the cells were treated with an inhibitor for an additional 24 h. Cells were subsequently harvested and lysed with lysis buffer [75 mM Tris–HCl, pH 6.8, 4% sodium dodecyl sulphate (SDS), 25% glycerol, 5% β-mercaptoethanol].

### Staining for tissues and cultured cells

For routine histological examination, the brains were fixed with 10% buffered formalin for 3 weeks. Blocks were cut from various cortical and subcortical regions, embedded in paraffin, sectioned, and then stained with hematoxylin and eosin. For immunohistochemistry, 4-μm-thick sections were cut from the frontal and temporal neocortex and hippocampus of patients with AD and controls. The sections were dehydrated and pretreated with 0.25% potassium permanganate for 15 min, then 2% oxalic acid for 3 min, followed by formic acid for 10 min for rabbit anti-P-S349 and anti-P-S403 antibodies. The sections were then subjected to immunohistochemical processing using the avidin-biotin-peroxidase complex method with diaminobenzidine (Sigma, Saint Louis, MO, USA). The sections were counterstained with hematoxylin.

Double immunofluorescent staining was performed to detect overlapping expression of phosphorylated p62 and phosphorylated tau or Keap1. For this purpose, the sections were blocked with donkey serum and then incubated overnight at 4°C with a mixture of rabbit anti-phosphorylated p62 and mouse anti-phosphorylated tau (AT8; Innogenetics, Ghent, Belgium) or rat anti-Keap1. On the next day, the sections were washed 5 times for 3 min each with PBS, followed by an incubation for 1 h with Alexa Fluor 488- and 594-conjugated secondary antibodies (Invitrogen). After a rinse with PBS, the sections were mounted with Fluoromount G (Southern Biotechnology Inc., Birmingham, AL, USA) and examined using a confocal microscope (EZ-Ci; Nikon, Tokyo, Japan). Adobe Photoshop CS5 software (Adobe systems, San Jose, CA, USA) was used for image processing.

After the cells were transfected with p62-EGFP wild-type or mutants for 24 h, cultured cells were double-immunolabelled with rabbit anti-phosphorylated p62 and mouse anti-p62 antibodies. Alexa Fluor 594- and 680-conjugated secondary antibodies (Invitrogen) were used.

### Fractionation of brain extracts

For biochemical analysis, brain tissues were dissected at autopsy and rapidly frozen at -70°C. Frozen tissues from the middle temporal cortex of patients with AD (n = 3) and control subjects (n = 3) were weighed and sequentially extracted with buffers of increasing detergent strength using a previously described protocol [24]. Briefly, samples were homogenised with 10 volumes of buffer A (10 mM Tris–HCl, pH 7.5, 1 mM EGTA, 10% sucrose, 0.8 M NaCl) and centrifuged (fraction 1). Afterwards, an equal volume of buffer A containing 2% Triton X-100 was added. The samples were then incubated for 30 min at 37°C and centrifuged at 100,000 × g for 30 min at 4°C (fraction 2). The resultant pellet was homogenised in 5 volumes of buffer A with 1% Sarkosyl and incubated for 30 min at 37°C. The homogenate was then centrifuged at 100,000 × g for 30 min at room temperature (fraction 3). The Sarkosyl-insoluble pellet was homogenised in 4 volumes of buffer A containing 1% 3-[(3-Cholamidopropyl) dimethylammonio] propanesulfonate (CHAPS) (Sigma) and centrifuged at 100,000 × g for 20 min at room temperature (fraction 4). The pellet was sonicated in 0.2 volumes of 8 M urea buffer (fraction 5). The CHAPS-soluble fraction (fraction 4) usually contains less total protein than other fractions by this method. We applied a constant volume of extract in each fraction to an SDS-polyacrylamide gel electrophoresis (PAGE).

### Immunoblot analysis

Frozen tissues form the middle temporal cortex of patients with AD (n = 8) and normal controls (n = 8) were used in this study. Each tissue was weighed and homogenised with a 20-fold volume of lysis buffer described above.

An equal amount of elution was subjected to SDS-PAGE, and Western blot analysis was performed as previously described [23]. Horseradish peroxidase-conjugated anti-mouse, anti-rat or anti-rabbit IgG (Santa Cruz Biotechnology, Santa Cruz, CA, USA) was used as a secondary antibody. Detection was performed according to the protocol provided with the ECL or ECL prime detection system (Amersham Pharmacia Biotech, Piscataway, NJ, USA). The data were quantified as described below and statistically analysed. Rabbit polyclonal antibodies against Keap1 (ProteinTech Group, Inc., Chicago, IL, USA), p62 (MBL, Nagoya, Japan), β-actin (Sigma), GFP (Invitrogen), acetylated histone (Millipore) and LC3 (Sigma), and mouse monoclonal antibodies against ubiquitin (FK1; Millipore) and Aβ oligomer (6E10; Covance, Richmond, CA, USA) were used in this study.

### Proteasomal activity assay

Chymotryptic, tryptic, and caspase proteasome activities were measured as described previously [25] with minor modifications. SH-SY5Y neuroblastoma cells were washed with PBS and pelleted by centrifugation. Cell pellets were sonicated in homogenisation buffer [25 mM Tris (pH 7.5), 100 mM NaCl, 5 mM ATP, 0.2% (v/v) NP-40 and 20% glycerol], and cell debris was removed by centrifugation at 4°C. Protein concentration in the resulting crude cellular extracts was determined by the bicinchoninic acid method (Pierce, Rockford, IL, USA). One hundred micrograms of protein from crude cellular extracts of each sample was diluted with buffer I [50 mM Tris–HCl (pH 7.4), 2 mM dithiothreitol, 5 mM MgCl_2_, 2 mM ATP] to a final volume of 0.5 mL (assayed in quadruplicate). Fluorogenic proteasome substrates were purchased from Boston Biochem (Cambridge, MA, USA): Suc-LLVY-7-amido-4-methylcoumarin (AMC) (chymotrypsin-like peptidase activity), Ac-RLR-AMC (trypsin-like peptidase activity), and Z-LLE-AMC (caspase-like or peptidylglutamyl peptide-hydrolysing activity). Each was dissolved in DMSO and brought to a final concentration of 80 μM. Proteolytic activities were assessed in 2 h at 25°C by measuring the release of the fluorescent group AMC using a fluorescence plate reader (Fluoroskan Ascent, Thermo Scientific, Waltham, MA, USA) with excitation and emission wavelengths of 380 and 460 nm, respectively.

### Dot blot analysis

We modified the filter-trap analysis as described previously [26]. Briefly, phosphorylated or non-phosphorylated peptides were diluted with TBS (Tris–HCl, pH 7.5, 150 mM NaCl), and applied to a 0.22-μm cellulose acetate membrane (Millipore) on a slot blot apparatus (Bio-Rad, Hercules, CA, USA) using a vacuum manifold. After washing with TBS containing 0.1% Triton X-100, the membrane was incubated with anti-phosphorylated p62 and pan-p62 antibodies and detected using the ECL detection system as described above.

### Quantitative analysis and statistical analysis

A semi-quantitative analysis of protein detection was performed by image analysis using the Image J software provided by the NIH. All of the values were represented as the means + standard deviation (SD). The statistical significance was evaluated using Student’s *t*-test when comparing two conditions. A probability value of less than 0.05 (p < 0.05) was considered to be significant.

## Results

### Specificity of antibodies against p62 phosphorylated at serine 349

**Figure 1:**
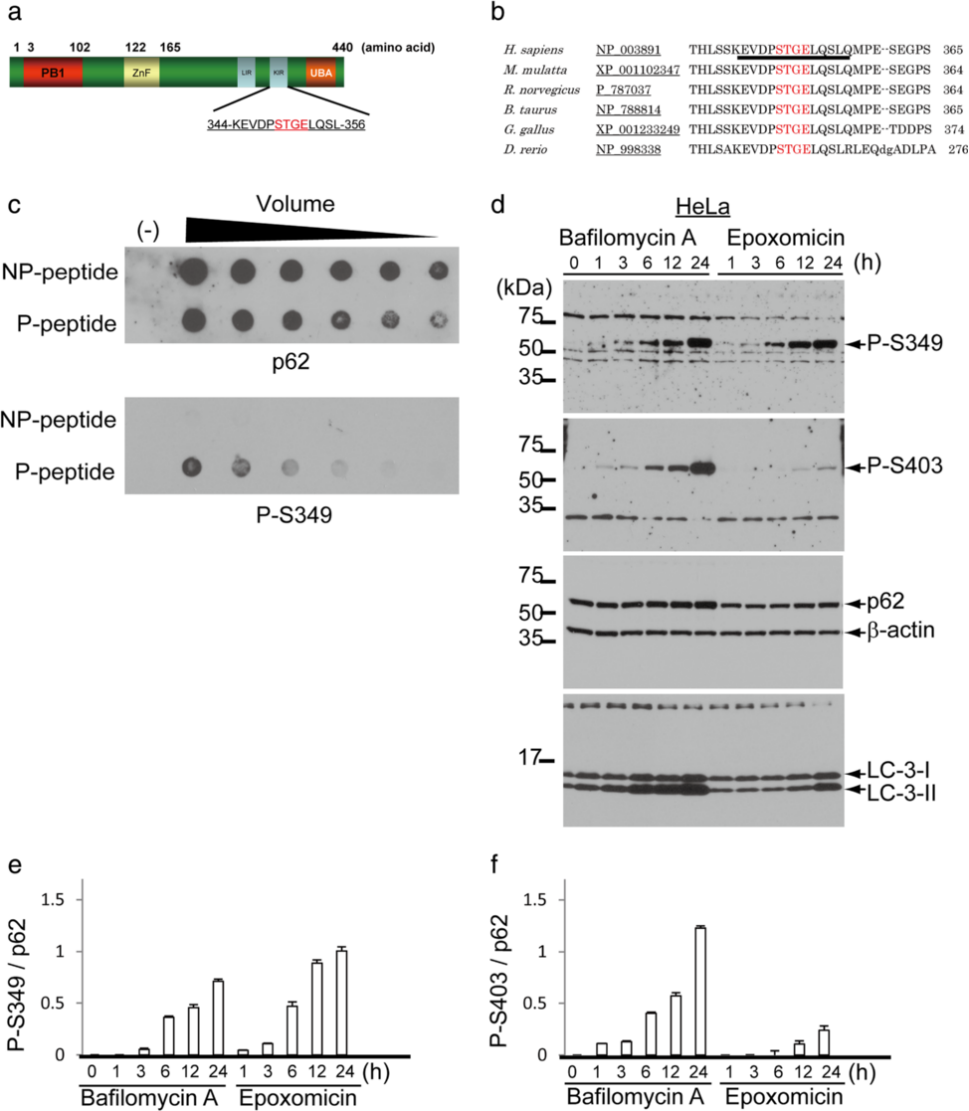
**Specificity of antibody against phosphorylated p62 at serine 349 (S349). (a)** Schematic representation of human p62. Number of amino acid is indicated. PB1, proteasomal binding region 1; ZnF, zinc finger; LIR, LC3-interacting region; KIR, Keap1-interacting region; UBA, ubiquitin associated domain. **(b)** Alignment of the KIR in p62 homologues. Underline represents the epitope for the antibody against phosphorylated p62. **(c)** Synthetic peptide (344–354 of human p62) was used to confirm the reactivity of phosphorylated p62-specific antibody by dot blot analysis. Phosphorylated peptide (P-peptide) is diluted, applied to a membrane and detected with the antibody against p62 phosphorylated at S349 (P-S349), whereas non-phosphorylated peptide (NP-peptide) is not detected. **(d)** HeLa cells were treated with an autophagic inhibitor, bafilomycin A (Baf), or a proteasome inhibitor, epoxomicin (Epo), for the indicated times. Two types of phosphorylation-specific antibodies differentially recognise p62 with phosphorylation on distinct residues. The molecular mass is indicated on the left side of the panel. **(e, f)** Quantitative data represents the ratio of the P-S349 **(e)** or P-S403 **(f)** level relative to the total p62 level at the indicated times following Baf or Epo treatment.

Recent papers reported that p62 binds to Keap1 through residues 344–356, called the Keap1-interacting region (KIR) (Figure 1a). This domain is conserved among species (Figure 1b), and STGE regions are very similar to the binding region (ETGE) of Nrf2 to Keap1. Because serine can be phosphorylated, this is potentially true of the serine within the STGE region of p62. To test this hypothesis, we raised antibodies against a synthetic peptide corresponding to amino acid residues 344–354 of human p62 with a phosphorylated S349 residue. After absorbance with the antibody against non-phosphorylated p62, ELISA and dot blot analysis confirmed that an antibody against phosphorylated p62 at S349 (referred to as P-S349) specifically recognised the phosphorylated peptide but not the non-phosphorylated peptide (Figure 1c). Similarly, a recent study demonstrated that S403 of p62 can be phosphorylated and that this phosphorylation strengthens the affinity with polyubiquitin and ubiquitinated molecules [22]. Therefore, we compared the reactivity of two antibodies against phosphorylated p62 (P-S349 and P-S403) in cultured cells treated with lysosome and proteasome inhibitors. In cells treated with a lysosome inhibitor (bafilomycin A), P-S349 and P-S403 signals increased in a time-dependent manner (Figure 1d). In contrast, after treatment with a proteasome inhibitor (epoxomicin), P-S349 signals were clearly detected in samples collected after 6 h, 12 h and 24 h, whereas P-S403 signals were faintly detected in samples collected after 12 h and 24 h. Quantitative intensity showed that the rate of the P-S349-positive signal to total p62 clearly increased in a time-dependent manner and that epoxomicin treatment more efficiently induced P-S349 than bafilomycin A (Figure 1e, f).

### S349 is phosphorylated following proteolysis disruption

**Figure 2:**
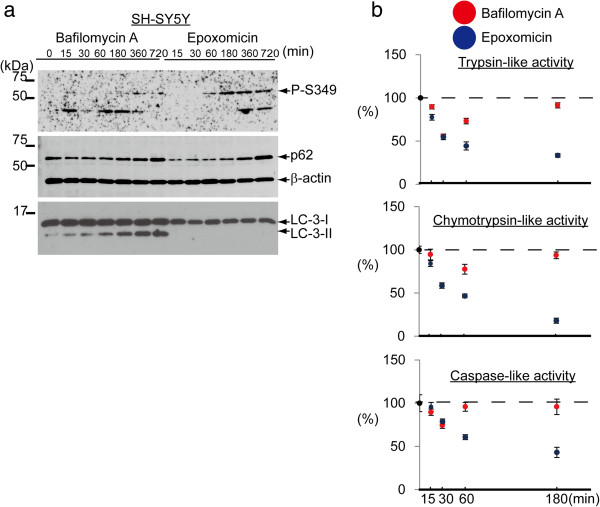
**The effect of proteasome inhibitor on p62 phosphorylation in a shorter time. (a)** SH-SY5Y cells were treated with Baf or Epo for the indicated times. Simultaneously, proteasomal activity was assessed. Immunoblotting analysis shows that both inhibitors induced the phosphorylation of S349 in a similar to HeLa cell. **(b)** A proteasomal activity assay shows that Epo inhibits trypsin-like, chymotrypsin-like and caspase-like activities of the proteasome in a time-dependent manner. While, Baf inhibits these activities in the first 30 minutes, and thereafter their activities returns to the basal levels.

We further investigated the effect of several proteasomal inhibitors on the phosphorylation of p62. P-S349 signals were also induced in neuroblastoma and HeLa cells following treatment with the proteasome inhibitors epoxomicin, MG132 or bortezomib for 24 h (Additional file 1: Figure S1). Immunoblotting showed that treatment with epoxomicin for 60 min faintly induced P-S349. Additionally, a proteasomal activity assay showed a significant decrease in trypsin-, chymotrypsin- and caspase-like activities in cells treated with epoxomicin in a time-dependent manner (Figure 2b).

**Figure 3:**
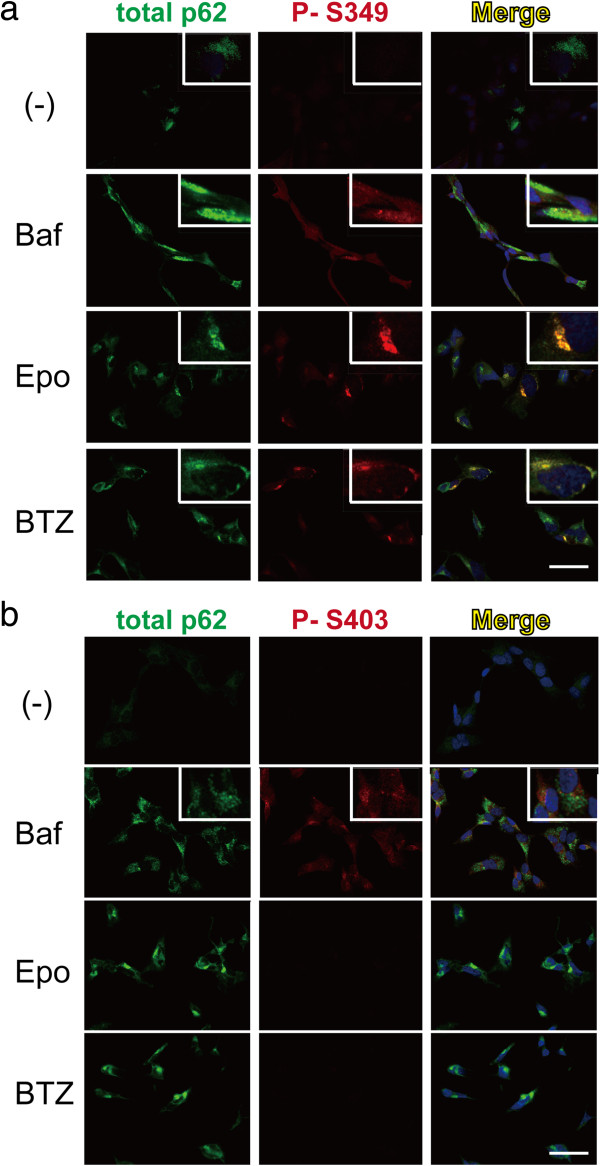
**The localisation of phosphorylated p62 in cells. (a)** The effect of autophagy or proteasomal inhibitor on the localisation of phosphorylated p62. Confocal immunofluorescence analysis shows that p62 immunoreactivity is weakly observed in cells without stimulation. After stimuli with autophagy or proteasome inhibitors, p62 is primarily observed in the cytoplasm (green) and forms cytoplasmic bodies. P-S349 on p62 (red) is undetectable in cells without stimulation. After Baf treatment for 24 h, P-S349 is observed throughout cells. In contrast, the P-S349 signal is concentrated in cytoplasmic bodies after treatment with the proteasome inhibitors Epo and bortezomib (BTZ). The islet panels indicate higher magnification. **(b)** After Baf treatment for 24 h, the P-S403 signal is enhanced. Proteasome inhibitors do not induce any signals of P-S403. Scale bar = 20 μm.

p62 has been reported to be phosphorylated at S266, T269 and S272, and these phosphorylations can change the cellular localisation of p62 [27]. We wanted to determine whether the phosphorylation of S349 can also contribute to the cellular localisation of p62. Immunofluorescence studies showed that p62 is weakly detected in the cytoplasm of untreated SH-SY5Y cells (Figure 3a) and that a positive signal for P-S349 was not detected in these cells. After treatment with an autophagy or proteasome inhibitor, p62 immunoreactivity was enhanced and cytoplasmic bodies formed. Consistent with the immunoblotting results, P-S349 signals were enhanced and densely accumulated in cytoplasmic inclusions in cells treated with the proteasome inhibitors epoxomicin and bortezomib (Figure 3a). In contrast, P-S403 signals were undetectable even in cytoplasmic inclusions (Figure 3b). In cells treated with bafilomycin A, P-S349 and P-S403 signals were broadly distributed throughout the cytoplasm of the cells. Similar results were observed in HeLa cells. Therefore, these data indicate that P-S349 localises to cytoplasmic bodies, and suggests that S349 phosphorylation is closely related to the formation or degradation of cytoplasmic inclusions after proteasomal disruption.

### S349 phosphorylation induces efficient formation of cytoplasmic inclusions

**Figure 4:**
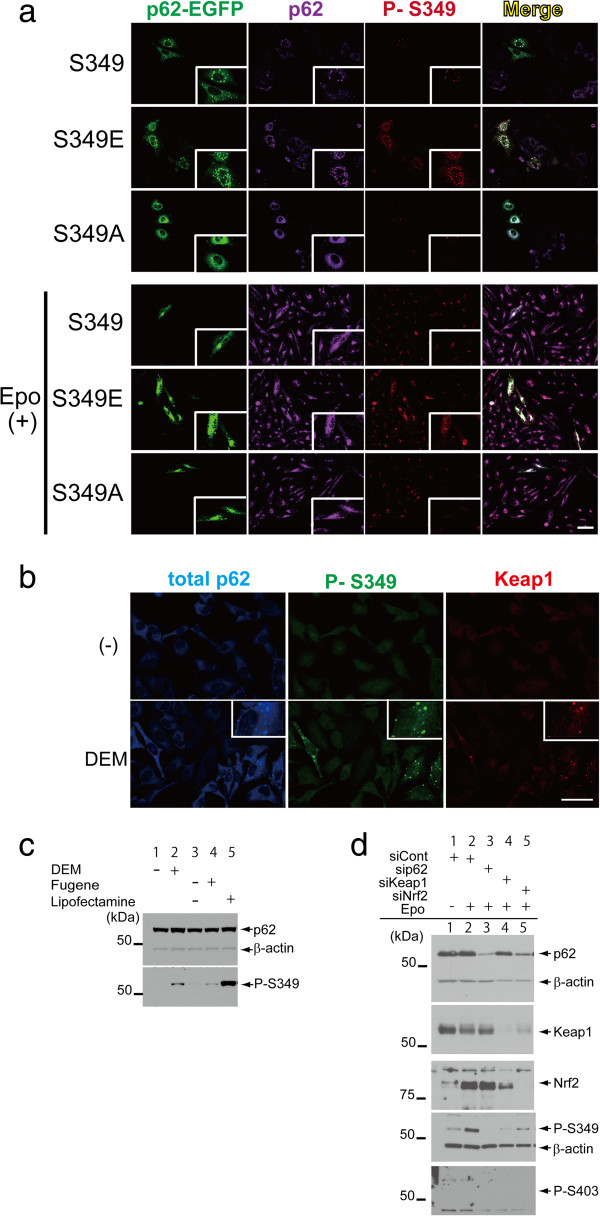
**The effect of phosphorylation-mimetic mutants on the formation of cytoplasmic inclusions in cells. (a)** p62 wild-type and mutants (S349E and S349A) fused with EGFP were transfected into HeLa cells. After 24 h, the cells were double-stained with rabbit anti-P-S349 (red) and mouse anti-pan-p62 (purple) antibodies. Wild-type p62 forms cytoplasmic inclusions in cells. The higher number of cytoplasmic inclusions is observed in cells transfected with the phosphorylation-mimetic mutant (S349E) compared with cells containing p62 wild-type and the S349A mutant (phosphorylation defective). An anti-phosphorylated p62 antibody reacts with S349E in inclusions, whereas this antibody only partially recognises inclusions in cells with p62 wild-type and S349A. Epo treatment increases the formation of S349 inclusions and p62 accumulation. The islet panels indicate higher magnification. Scale bar = 20 μm. **(b)** DEM also enhances the formation of p62 (blue) and phosphorylation of S349 (green). Keap1 (red) also colocalised with P-S349 signals. The islet panels indicate higher magnification. Scale bar = 20 μm. **(c)** Cells were treated with or without DEM for 24 h, and with transfection reagent alone (Fugene 6 or Lipofectamine 2000). Cells were harvested and analysed by immunoblotting. P-S349 signals are detected in samples treated with DEM (lane 2) and transfection reagents (lanes 4 and 5). **(d)** Knockdown of p62, Keap1 or Nrf2 was performed using Lipofectamine RNAi MAX. After 24 h, cells were treated with Epo for 24 h and harvested. Epo treatment enhances the P-S349 signal in cells treated with control siRNA compared to vehicle alone. No signals for P-S349 are observed in cells subjected to siRNA against p62 and siKeap1. Similar to other transfection reagents, Lipofectamine RNAi MAX slightly induced the phosphorylation of p62 at S349 (lane 1).

To clarify the role of P-S349 in the formation of cytoplasmic inclusions, we compared wild-type p62 with a phosphorylation-mimetic (S349E) or a phosphorylation-defective (S349A) mutant. Immunocytofluorescence studies showed that the S349E mutant caused a higher number of cytoplasmic inclusions compared with p62 wild-type and the S349A mutant (Figure 4a). The anti-P-S349 antibody strongly detected the p62 S349E mutant, but only weakly detected p62 wild-type and the S349A mutant. This result was consistent with the result of immunoblotting (Additional file 2: Figure S2). Epoxomicin treatment increased this reaction and induced aggresomes, which are localised near the nucleus [28]. Aggresomes were immunostained with histone deacetylase 6 antibody and were also positive for P-S349 (data not shown), suggesting that P-S349 enhances inclusion formation. Importantly, oxidative stress also induced P-S349. We used DEM as a stress inducer, which clearly induced the formation of cytoplasmic inclusions and P-S349 signal (Figure 4b). In addition, Keap1 colocalised with P-S349 in the inclusions. During these experiments, we observed that p62 is phosphorylated at S349 after transfections using Fugene 6 and Lipofectamine 2000 (Additional file 3: Figure S3). These results were consistent with the immunoblotting showing that Lipofectamine 2000 clearly induced phosphorylation, and DEM and Fugene 6 moderately induced phosphorylation of p62 at S349 (Figure 4c).

We next examined the effect of disruption of the p62-Keap1-Nrf2 complex on the phosphorylation of p62 (Figure 4d). Epoxomicin treatment enhanced the P-S349 signal in cells treated with control siRNA, whereas no P-S349 signal was observed in cells subjected to sip62 or siKeap1, and a weaker signal in cells treated with siNrf2 compared to controls. Knockdown experiments using siRNA suggested that this phosphorylation is sensitive to alteration of the tertiary structure of the p62-Keap1 complex. Collectively, these results indicate that p62 is phosphorylated at S349 under several pathological conditions and that phosphorylation at S349 efficiently forms cytoplasmic inclusions, leading to a higher affinity for Keap1.

### S349 is phosphorylated in the brains with alzheimer’s disease

**Figure 5:**
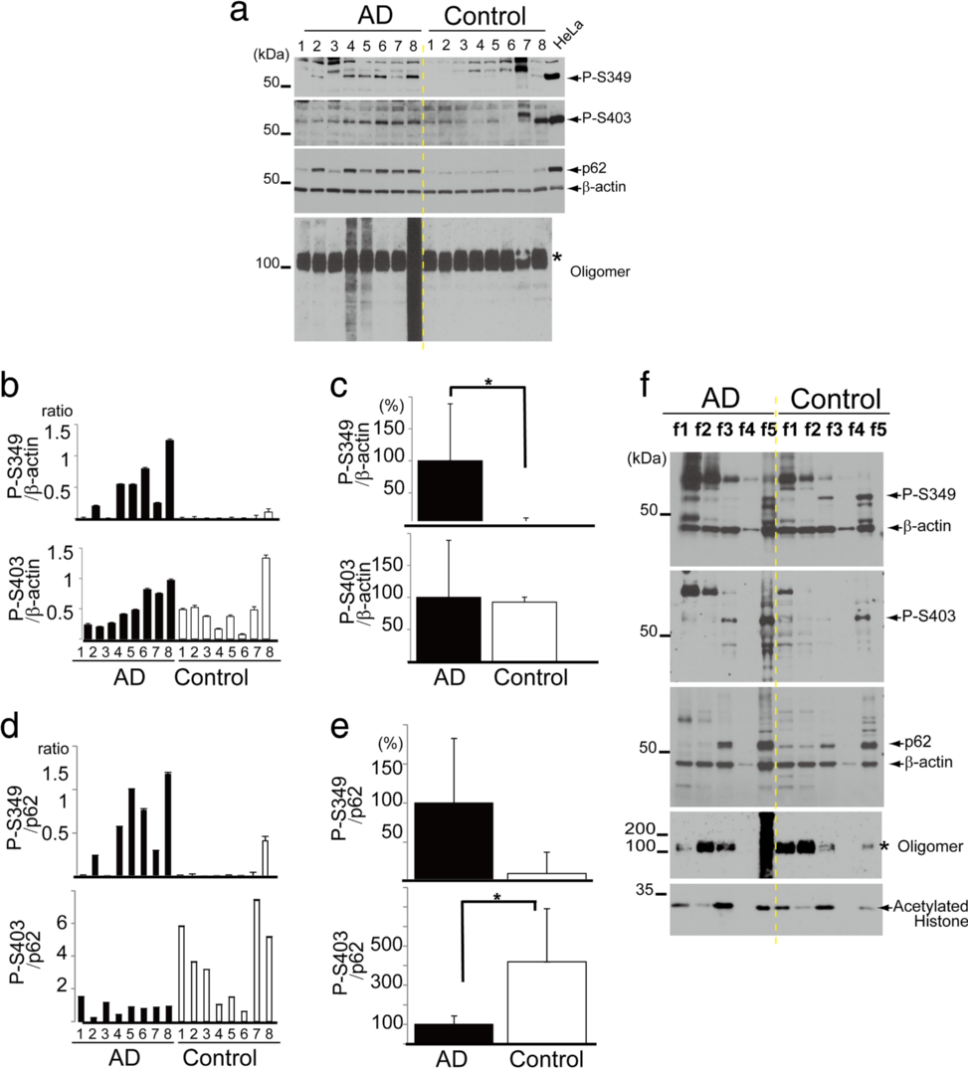
**Phosphorylation of p62 in the brains of patients with AD. (a)** Frozen tissues (the temporal cortex) of patients with AD and controls were lysed and subjected to SDS-PAGE. HeLa cells were treated with Baf, and used as a positive control. P-S349 is detected in almost all patients with AD but is only faintly detected in controls. The P-S403 signal is visible in all samples except for a control subject (case 6). An anti-oligomer antibody detects smear bands in some patients with AD. The approximately 100 kDa bands appear to be non-specific (*). **(b)** The ratio of P-S349 or P-S403 levels against β-actin was measured. **(c)** Quantitative data show that the level of P-S349, but not of P-S403, is significantly increased in AD compared with controls (*p < 0.01). The value in AD is defined as 100%. **(d)** The ratio of P-S349 or P-S403 levels against p62 was measured. **(e)** The level of P-S403, but not of P-S349, is significantly decreased in AD compared with controls (*p < 0.05). **(f)** Frozen tissues were obtained from the temporal cortex of patients with AD and control subjects (n = 3 in each group). Sequential biochemical fractionation shows that P-S349 is detected in fractions 1 and 5 (f1 and f5) in AD, and f3 and f5 in controls. Note that the signal is enhanced in f1 in AD samples compared with controls. Instead, the signal is weaker in f5 of controls than that of AD samples. P-S403 is distributed in f1, f3 and f5 in AD, and f1 and f5 in controls. Similar results are observed in other samples of AD and control cases. The CHAPS-soluble fraction (f4) has a lower quantity of proteins compared with other fractions. Acetylated histone is used as one of the nuclear proteins. Non-specific (*).

We previously demonstrated that p62 and Keap1 are colocalised in the cytoplasmic inclusions observed in the brains of patients with neurodegenerative dementia (neurofibrillary tangles in AD, and Lewy bodies in DLB [10]). Additionally, higher levels of p62 are observed in AD compared to controls. Therefore, we next determined whether phosphorylation of S349 and S403 occurs in the brains of patients with AD and control subjects using antibodies against phosphorylated p62. Immunoblot analysis showed that anti-P-S349 and anti-P-S403 antibodies recognised signals in the brains similar to those observed in cultured cells with an inhibitor (Figure 5a). Notably, the signal intensity of P-S349 was higher in AD compared with controls (Figure 5b), and quantitative data showed that the ratio of the P-S349 level to actin was increased in AD relative to controls (Figure 5c). The S403 level was unchanged between AD and controls. Quantitative data showed that the ratio of the P-S349 level to total p62 was also increased in AD relative to controls (Figure 5d, e). The S403 level was repressed in AD relative to controls.

Further studies were performed to determine whether the phosphorylation of p62 alters its solubility. Frozen samples were fractionated with detergents of increasing strength and subsequently analysed by immunoblotting. The P-S349 signal was primarily detected in detergent-insoluble fractions (fraction 5) in controls and AD (top panel in Figure 5f). In addition, detergent-soluble P-S349 was also present in fraction 3 in controls. However, it was shifted to fraction 1 in AD. P-S403 signal was distributed to fractions 1, 3 and 5 in AD and fractions 1 and 5 in controls (the second top panel in Figure 5f). Because total p62 is primarily detected in fractions 3 and 5 in controls and AD (the third top panel in Figure 5f), this suggests that P-S349 is easily solubilised and its level is increased in AD brains.

**Figure 6:**
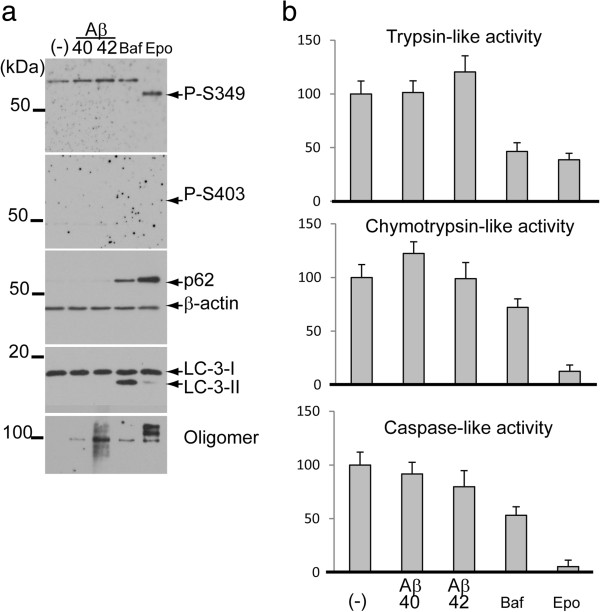
**The effect of amyloid** **β** **(A** **β** **) on phosphorylated p62 levels. (a)** SH-SY5Y neuroblastoma cells were treated with Aβ40 or Aβ42, Baf or Epo. After 24 h, cells were harvested, lysed and analysed by immunoblotting. Epo increases the levels of total p62 and P-S349, whereas the P-S403 level is undetectable. In a sample treated with Baf, lipidated LC3 (LC3-II) is induced in a time-dependent manner, but signals for P-S349 and P-S403 are undetectable due to the low level of total p62. **(b)** A proteasomal activity assay confirms that Epo inhibits trypsin-like, chymotrypsin-like and caspase-like activities of the proteasome. Baf mildly inhibits these activities. However, Aβ40 and Aβ42 have no effect on proteasome activity and autophagic flux.

Based on these results, we hypothesise that an excess of Aβ potentially influences the phosphorylation of p62 because Aβ can induce tau phosphorylation [29, 30]. To test this hypothesis, SH-SY5Y cells were treated with Aβ40 or Aβ42 for 24 h. Although the levels of total p62 and an autophagosomal marker protein (LC3-II) were increased following disruption of the protein degradation systems (Figure 6a), Aβ40 or Aβ42 did not induce phosphorylation of p62 at S349 and S403. We further treated cells with Aβ for an additional 48 h or 72 h. However, no phosphorylation was induced (data not shown). The proteasomal activity assay showed that epoxomicin strongly inhibited trypsin-like, chymotrypsin-like and caspase-like activities of the proteasome (Figure 6b). Bafilomycin A mildly inhibited trypsin-like activity. Aβ40 and Aβ42 did not have any effect on proteasomal and autophagic activities.

### Neurofibrillary tangles are positive for P-S349

**Figure 7:**
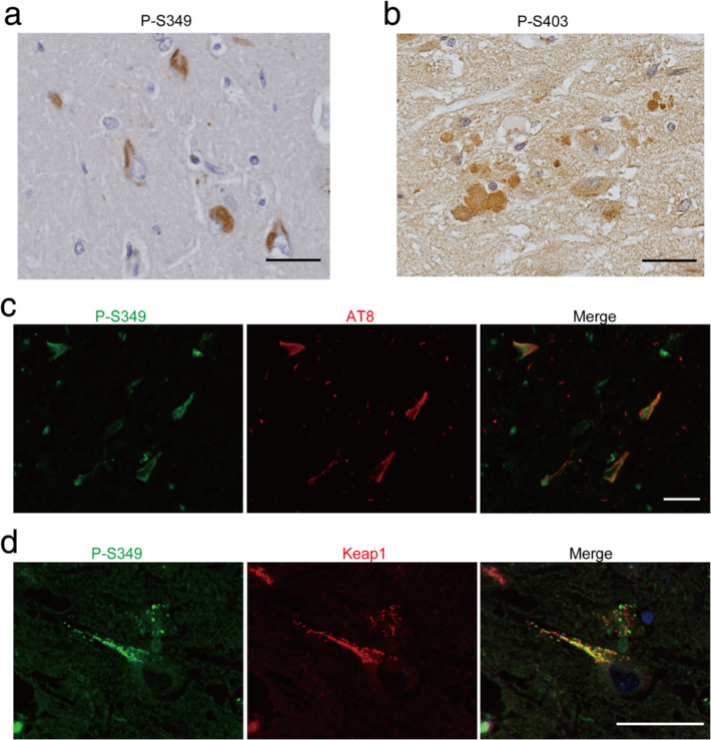
**Immunohistochemical localisation of P-S349 and P-S403 in the brains of patients with AD. (a)** Neurofibrillary tangles in the entorhinal cortex. **(b)** Dystrophic neurites of senile plaques. **(c)** Colocalisation of P-S349 (green) and phosphorylated tau (red) in neurofibrillary tangles. Bars = 50 μm. **(d)** Colocalisation of P-S349 (green) and Keap1 (red) in neurofibrillary tangles. Bars = 20 μm.

To investigate where P-S349 is localised in AD brains, we performed immunohistochemical studies. The anti-P-S349 antibody strongly immunolabelled neurofibrillary tangles (NFTs) in AD (Figure 7a). Positive signals in NFTs were abolished by alkaline phosphatase treatment. In addition, absorption of the anti-P-S349 antibody with phosphorylated peptide diminished the staining. Alternatively, the anti-P-S403 antibody weakly or moderately stained dystrophic neurites in amyloid plaques (Figure 7b). The anti-P-S403 antibody did not react with NFTs. No immunoreactivity was observed in control sections using both antibodies. A double-immunofluorescence study confirmed the colocalisation of P-S349 and phosphorylated tau (Figure 7c) or Keap1 (Figure 7d).

## Discussion

Under oxidative stress and certain pathological conditions, the binding affinity of Keap1 for Nrf2 is decreased, leading to the intranuclear shuttling of Nrf2 and the subsequent increase of several types of genes, including antioxidant and detoxifying enzymes. In our study, exposure to oxidative stress (DEM) induced the phosphorylation of p62 at S349 within KIR, which is the binding region for Keap1. Total p62 levels are known to increase by oxidative stress [31]. Because the phosphorylation of S349 on p62 strengthens its affinity for Keap1 [20, 21], higher p62 levels and an increased affinity for Keap1 simultaneously occur under oxidative stress. In addition, we showed that disruption of protein degradation systems can also induce the phosphorylation of S349. Although S403 on p62 is also known to be phosphorylated by inhibition of autophagic flux, proteasome inhibition had less of an effect on the phosphorylation of S403 than on S349. This result was also supported by immunofluorescence showing that P-S349 signals were clearly observed in the cytoplasmic inclusions of cultured cells. In contrast, P-S403 signals were weakly detected in cytoplasmic inclusions. Because the phosphorylation of S403 likely enhances the affinity to ubiquitinated molecules and helps degrade them by autophagy, it is possible that P-S403 signals physiologically occur, but may be relatively undetectable due to rapid self-degradation. By contrast, p62 S394 is efficiently phosphorylated in response to proteasome inhibition and exposure to oxidative stress. Furthermore, even transfection reagents can induce P-S394 immunoreactivity. These data suggest that the ratio of the P-S394 level to total p62 may be an indicator for noxious stimuli to cells.

Phosphorylation-mimetic mutations have been commonly used to investigate the role of phosphorylation. Our specific antibody against P-S349 recognised the phosphorylation-mimetic mutant p62 S349E. Immunofluorescence showed that the S349E mutant had a higher number of inclusions than p62 wild-type and S349A, the latter being a phosphorylation-deficient mutant. An anti-phosphorylated p62 antibody strongly detected signals in cells with S349E, whereas it showed partial reactivity in cellular inclusions with p62 wild-type and S349A. Thus, it is likely that the phosphorylation of S349 enhances the formation of inclusions. Because Nrf2 target genes are activated through the higher affinity of Keap1 for p62 with P-S349, inclusion formation itself exerts cyto-protective roles in the early stages of this process.

Importantly, we showed that S349 on p62 is in fact phosphorylated in the brains of patients with AD and that the level was significantly increased in AD compared with controls. Consistent with previous results [9, 32], we also demonstrated that the total p62 level was significantly increased in AD relative to controls. The ratio of P-S349 levels to total p62 is significantly increased in AD relative to controls. This suggests a disruption of protein degradation systems and/or the occurrence of oxidative stress in the brains with AD, and the data also support previous evidence demonstrating decreased proteasome activity [11–13] and sustained oxidative stress occur in AD compared with controls [33–35].

On the basis of these findings, we hypothesise that Aβ deposition can induce phosphorylation of S349. Unexpectedly, however, we failed to find any significant changes induced by Aβ40 or Aβ42 in our system. Anti-Aβ oligomer antibody clearly showed positive signals in cells treated with Aβ42, but not with Aβ40, and we confirmed that only Aβ42 was accumulated in lysosomes using confocal microscopy as previously described [36–38]. Therefore, our experimental system works, but further studies will be needed to clarify the relationship between Aβ deposition, phosphorylation, and protein degradation using primary neuronal cells or an in vivo system.

p62 is physiologically expressed in most organs, and can be incorporated into a wide spectrum of ubiquitin-positive inclusions in neurodegenerative diseases [39–41]. In AD, p62 is extensively accumulated in NFTs [40]. Similarly, we showed that an anti-P-S349 antibody specifically reacted with NFTs, whereas an anti-P-S403 antibody recognised dystrophic neurites in amyloid plaques. Both pathological lesions (NFTs and dystrophic neurites) are composed of hyper-phosphorylated tau. Babu et al. reported that p62 interacts with ubiquitinated tau through its UBA domain and helps degrade tau by a ubiquitin-proteasome process [42]. As described above, phosphorylated p62 at S403 is highly involved in the degradation of ubiquitinated molecules by the autophagy-lysosome system [22]. Taken together, phosphorylated tau is potentially delivered to the proteasome as well as the autophagy-lysosome system together with p62. Importantly, NFTs were immunonegative for P-S403. These results suggest that P-S349 or P-S403 on p62 is able to distinguish hyper-phosphorylated tau in NFTs from that in dystrophic neurites.

## Conclusion

In conclusion, we provide evidence that S349 on p62 is efficiently phosphorylated following inhibition of the proteasome and of autophagy. Because p62 is highly involved in protein degradation systems as well as the pathogenesis of several diseases, these results suggest that post-translational modifications of p62 have pivotal roles in the progression of neurodegeneration in AD.

## Electronic supplementary material

Additional file 1: Figure S1: SH-SY5Y cells were treated with Epo, MG132 (MG), and Lactacysin (Lac) for the indicated hours. Immunoblotting analysis demonstrates that all treatments induced phosphorylation of S349. LC3-II level is gradually increased in cells treated with proteasome inhibitors. (TIFF 977 KB)

Additional file 2: Figure S2: Reactivity of P-S349 antibody for mutated p62. Transfection with p62-EGFP (wild type, phosphorylation-mimetic or defective mutants) was performed and analysed by immunoblotting. P-S349 antibody reacts with p62-S349E and wild type. Additionally, endogenous p62 is slightly phosphorylated in cells with p62-S349E and wild type, but not p62-S349A. Anti-GFP antibody recognises p62-EGFP and mutants. Anti-p62 antibody reacts with endogenous p62 as well as p62-EGFP and mutants. (TIFF 929 KB)

Additional file 3: Figure S3: The effect of transient transfection on p62 phosphorylation. HeLa cells were treated with transfection reagent alone, Fugene 6 or Lipofectamine 2000 (Lipo) for 24 h. Confocal immunofluorescence analysis shows that total p62 (blue) forms cytoplasmic bodies, and P-S349 signals (green) are detected in these inclusions. Endogenous Keap1 (red) is weakly detected in the cytoplasm. The Keap1 signal is enhanced in cells transfected with Lipo and is partially colocalised in p62 inclusions. The islet panels indicate higher magnification. Scale bar = 20 μm. (TIFF 3 MB)

## References

[1] Bedford L, Hay D, Devoy A, Paine S, Powe DG, Seth R, Gray T, Topham I, Fone K, Rezvani N, Mee M, Soane T, Layfield R, Sheppard PW, Ebendal T, Usoskin D, Lowe J, Mayer RJ (2008). Depletion of 26S proteasomes in mouse brain neurons causes neurodegeneration and Lewy-like inclusions resembling human pale bodies. J Neurosci.

[2] Komatsu M, Waguri S, Chiba T, Murata S, Iwata J, Tanida I, Ueno T, Koike M, Uchiyama Y, Kominami E, Tanaka K (2006). Loss of autophagy in the central nervous system causes neurodegeneration in mice. Nature.

[3] Bjorkoy G, Lamark T, Brech A, Outzen H, Perander M, Overvatn A, Stenmark H, Johansen T (2005). p62/SQSTM1 forms protein aggregates degraded by autophagy and has a protective effect on huntingtin-induced cell death. J Cell Biol.

[4] Johansen T, Lamark T (2011). Selective autophagy mediated by autophagic adapter proteins. Autophagy.

[5] Seibenhener ML, Babu JR, Geetha T, Wong HC, Krishna NR, Wooten MW (2004). Sequestosome 1/p62 is a polyubiquitin chain binding protein involved in ubiquitin proteasome degradation. Mol Cell Biol.

[6] Pankiv S, Clausen TH, Lamark T, Brech A, Bruun JA, Outzen H, Overvatn A, Bjorkoy G, Johansen T (2007). p62/SQSTM1 binds directly to Atg8/LC3 to facilitate degradation of ubiquitinated protein aggregates by autophagy. J Biol Chem.

[7] Kabeya Y, Mizushima N, Yamamoto A, Oshitani-Okamoto S, Ohsumi Y, Yoshimori T (2004). LC3, GABARAP and GATE16 localize to autophagosomal membrane depending on form-II formation. J Cell Sci.

[8] Komatsu M, Waguri S, Koike M, Sou YS, Ueno T, Hara T, Mizushima N, Iwata J, Ezaki J, Murata S, Hamazaki J, Nishito Y, Iemura S, Natsume T, Yanagawa T, Uwayama J, Warabi E, Yoshida H, Ishii T, Kobayashi A, Yamamoto M, Yue Z, Uchiyama Y, Kominami E, Tanaka K (2007). Homeostatic levels of p62 control cytoplasmic inclusion body formation in autophagy-deficient mice. Cell.

[9] Odagiri S, Tanji K, Mori F, Kakita A, Takahashi H, Wakabayashi K (2012). Autophagic adapter protein NBR1 is localized in Lewy bodies and glial cytoplasmic inclusions and is involved in aggregate formation in alpha-synucleinopathy. Acta Neuropathol.

[10] Tanji K, Maruyama A, Odagiri S, Mori F, Itoh K, Kakita A, Takahashi H, Wakabayashi K (2013). Keap1 is localized in neuronal and glial cytoplasmic inclusions in various neurodegenerative diseases. J Neuropathol Exp Neurol.

[11] Sayre LM, Zelasko DA, Harris PL, Perry G, Salomon RG, Smith MA (1997). 4-Hydroxynonenal-derived advanced lipid peroxidation end products are increased in Alzheimer’s disease. J Neurochem.

[12] Smith MA, Kutty RK, Richey PL, Yan SD, Stern D, Chader GJ, Wiggert B, Petersen RB, Perry G (1994). Heme oxygenase-1 is associated with the neurofibrillary pathology of Alzheimer’s disease. Am J Pathol.

[13] Valko M, Leibfritz D, Moncol J, Cronin MT, Mazur M, Telser J (2007). Free radicals and antioxidants in normal physiological functions and human disease. Int J Biochem Cell Biol.

[14] Copple IM, Lister A, Obeng AD, Kitteringham NR, Jenkins RE, Layfield R, Foster BJ, Goldring CE, Park BK (2010). Physical and functional interaction of sequestosome 1 with Keap1 regulates the Keap1-Nrf2 cell defense pathway. J Biol Chem.

[15] Jain A, Lamark T, Sjottem E, Larsen KB, Awuh JA, Overvatn A, McMahon M, Hayes JD, Johansen T (2010). p62/SQSTM1 is a target gene for transcription factor NRF2 and creates a positive feedback loop by inducing antioxidant response element-driven gene transcription. J Biol Chem.

[16] Komatsu M, Kurokawa H, Waguri S, Taguchi K, Kobayashi A, Ichimura Y, Sou YS, Ueno I, Sakamoto A, Tong KI, Kim M, Nishito Y, Iemura S, Natsume T, Ueno T, Kominami E, Motohashi H, Tanaka K, Yamamoto M (2010). The selective autophagy substrate p62 activates the stress responsive transcription factor Nrf2 through inactivation of Keap1. Nat Cell Biol.

[17] Lau A, Wang XJ, Zhao F, Villeneuve NF, Wu T, Jiang T, Sun Z, White E, Zhang DD (2010). A noncanonical mechanism of Nrf2 activation by autophagy deficiency: direct interaction between Keap1 and p62. Mol Cell Biol.

[18] Itoh K, Wakabayashi N, Katoh Y, Ishii T, Igarashi K, Engel JD, Yamamoto M (1999). Keap1 represses nuclear activation of antioxidant responsive elements by Nrf2 through binding to the amino-terminal Neh2 domain. Genes Dev.

[19] Liu Y, Kern JT, Walker JR, Johnson JA, Schultz PG, Luesch H (2007). A genomic screen for activators of the antioxidant response element. Proc Natl Acad Sci U S A.

[20] Hancock R, Bertrand HC, Tsujita T, Naz S, El-Bakry A, Laoruchupong J, Hayes JD, Wells G (2012). Peptide inhibitors of the Keap1-Nrf2 protein-protein interaction. Free Radic Biol Med.

[21] Ichimura Y, Waguri S, Sou YS, Kageyama S, Hasegawa J, Ishimura R, Saito T, Yang Y, Kouno T, Fukutomi T, Hoshii T, Hirao A, Takagi K, Mizushima T, Motohashi H, Lee MS, Yoshimori T, Tanaka K, Yamamoto M, Komatsu M (2013). Phosphorylation of p62 activates the Keap1-Nrf2 pathway during selective autophagy. Mol Cell.

[22] Matsumoto G, Wada K, Okuno M, Kurosawa M, Nukina N (2011). Serine 403 phosphorylation of p62/SQSTM1 regulates selective autophagic clearance of ubiquitinated proteins. Mol Cell.

[23] Tanji K, Zhang HX, Mori F, Kakita A, Takahashi H, Wakabayashi K (2012). p62/sequestosome 1 binds to TDP-43 in brains with frontotemporal lobar degeneration with TDP-43 inclusions. J Neurosci Res.

[24] Tanji K, Kamitani T, Mori F, Kakita A, Takahashi H, Wakabayashi K (2010). TRIM9, a novel brain-specific E3 ubiquitin ligase, is repressed in the brain of Parkinson’s disease and dementia with Lewy bodies. Neurobiol Dis.

[25] Lee BH, Lee MJ, Park S, Oh DC, Elsasser S, Chen PC, Gartner C, Dimova N, Hanna J, Gygi SP, Wilson SM, King RW, Finley D (2010). Enhancement of proteasome activity by a small-molecule inhibitor of USP14. Nature.

[26] Tanji K, Tanaka T, Mori F, Kito K, Takahashi H, Wakabayashi K, Kamitani T (2006). NUB1 suppresses the formation of Lewy body-like inclusions by proteasomal degradation of synphilin-1. Am J Pathol.

[27] Pankiv S, Lamark T, Bruun JA, Overvatn A, Bjorkoy G, Johansen T (2010). Nucleocytoplasmic shuttling of p62/SQSTM1 and its role in recruitment of nuclear polyubiquitinated proteins to promyelocytic leukemia bodies. J Biol Chem.

[28] Olzmann JA, Li L, Chudaev MV, Chen J, Perez FA, Palmiter RD, Chin LS (2007). Parkin-mediated K63-linked polyubiquitination targets misfolded DJ-1 to aggresomes via binding to HDAC6. J Cell Biol.

[29] Busciglio J, Lorenzo A, Yeh J, Yankner BA (1995). beta-amyloid fibrils induce tau phosphorylation and loss of microtubule binding. Neuron.

[30] Sturchler-Pierrat C, Abramowski D, Duke M, Wiederhold KH, Mistl C, Rothacher S, Ledermann B, Burki K, Frey P, Paganetti PA, Waridel C, Calhoun ME, Jucker M, Probst A, Staufenbiel M, Sommer B (1997). Two amyloid precursor protein transgenic mouse models with Alzheimer disease-like pathology. Proc Natl Acad Sci U S A.

[31] Ishii T, Yanagawa T, Kawane T, Yuki K, Seita J, Yoshida H, Bannai S (1996). Murine peritoneal macrophages induce a novel 60-kDa protein with structural similarity to a tyrosine kinase p56lck-associated protein in response to oxidative stress. Biochem Biophys Res Commun.

[32] Bartlett BJ, Isakson P, Lewerenz J, Sanchez H, Kotzebue RW, Cumming RC, Harris GL, Nezis IP, Schubert DR, Simonsen A, Finley KD (2011). p62, Ref (2) P and ubiquitinated proteins are conserved markers of neuronal aging, aggregate formation and progressive autophagic defects. Autophagy.

[33] Keller JN, Hanni KB, Markesbery WR (2000). Impaired proteasome function in Alzheimer’s disease. J Neurochem.

[34] Lam YA, Pickart CM, Alban A, Landon M, Jamieson C, Ramage R, Mayer RJ, Layfield R (2000). Inhibition of the ubiquitin-proteasome system in Alzheimer’s disease. Proc Natl Acad Sci U S A.

[35] Lopez Salon M, Morelli L, Castano EM, Soto EF, Pasquini JM (2000). Defective ubiquitination of cerebral proteins in Alzheimer’s disease. J Neurosci Res.

[36] Hu X, Crick SL, Bu G, Frieden C, Pappu RV, Lee JM (2009). Amyloid seeds formed by cellular uptake, concentration, and aggregation of the amyloid-beta peptide. Proc Natl Acad Sci U S A.

[37] Nixon RA, Cataldo AM, Mathews PM (2000). The endosomal-lysosomal system of neurons in Alzheimer’s disease pathogenesis: a review. Neurochem Res.

[38] Yang AJ, Chandswangbhuvana D, Margol L, Glabe CG (1998). Loss of endosomal/lysosomal membrane impermeability is an early event in amyloid Abeta1-42 pathogenesis. J Neurosci Res.

[39] Arai T, Nonaka T, Hasegawa M, Akiyama H, Yoshida M, Hashizume Y, Tsuchiya K, Oda T, Ikeda K (2003). Neuronal and glial inclusions in frontotemporal dementia with or without motor neuron disease are immunopositive for p62. Neurosci Lett.

[40] Kuusisto E, Salminen A, Alafuzoff I (2001). Ubiquitin-binding protein p62 is present in neuronal and glial inclusions in human tauopathies and synucleinopathies. Neuroreport.

[41] Nakano T, Nakaso K, Nakashima K, Ohama E (2004). Expression of ubiquitin-binding protein p62 in ubiquitin-immunoreactive intraneuronal inclusions in amyotrophic lateral sclerosis with dementia: analysis of five autopsy cases with broad clinicopathological spectrum. Acta Neuropathol.

[42] Babu JR, Geetha T, Wooten MW (2005). Sequestosome 1/p62 shuttles polyubiquitinated tau for proteasomal degradation. J Neurochem.

