# Supplementary figures and images for: Phosphorylation of serine 349 of p62 in Alzheimer’s disease brain
Source: Acta Neuropathol Commun. 2014 May 3;2:50. doi: 10.1186/2051-5960-2-50 (PMC4035093; doi:10.1186/2051-5960-2-50)

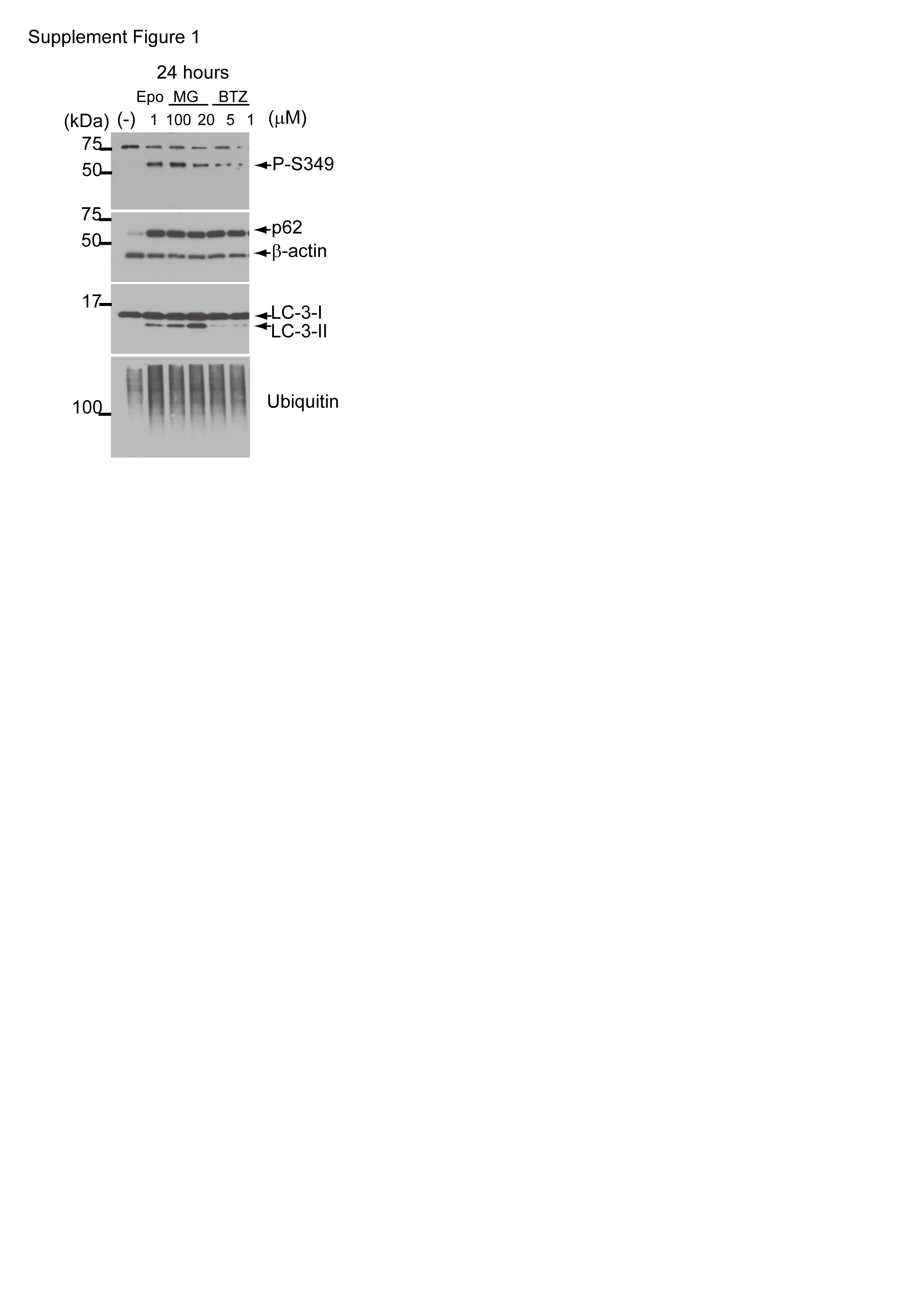

Supplement: Supplementary file 1 — Additional file 1: Figure S1: SH-SY5Y cells were treated with Epo, MG132 (MG), and Lactacysin (Lac) for the indicated hours. Immunoblotting analysis demonstrates that all treatments induced phosphorylation of S349. LC3-II level is gradually increased in cells treated with proteasome inhibitors. (TIFF 977 KB) [file 40478_2014_124_MOESM1_ESM.tiff]

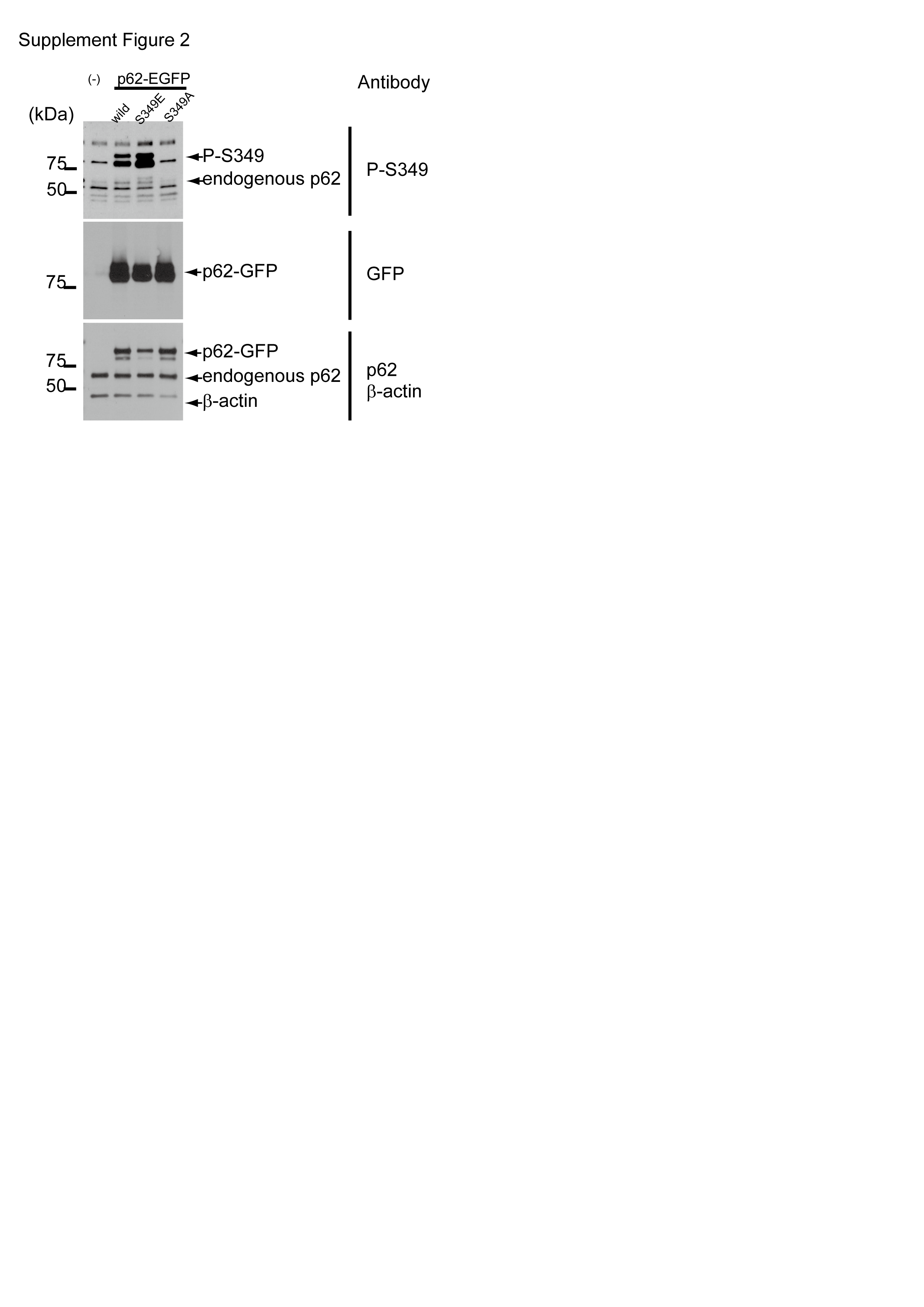

Supplement: Supplementary file 2 — Additional file 2: Figure S2: Reactivity of P-S349 antibody for mutated p62. Transfection with p62-EGFP (wild type, phosphorylation-mimetic or defective mutants) was performed and analysed by immunoblotting. P-S349 antibody reacts with p62-S349E and wild type. Additionally, endogenous p62 is slightly phosphorylated in cells with p62-S349E and wild type, but not p62-S349A. Anti-GFP antibody recognises p62-EGFP and mutants. Anti-p62 antibody reacts with endogenous p62 as well as p62-EGFP and mutants. (TIFF 929 KB) [file 40478_2014_124_MOESM2_ESM.tiff]

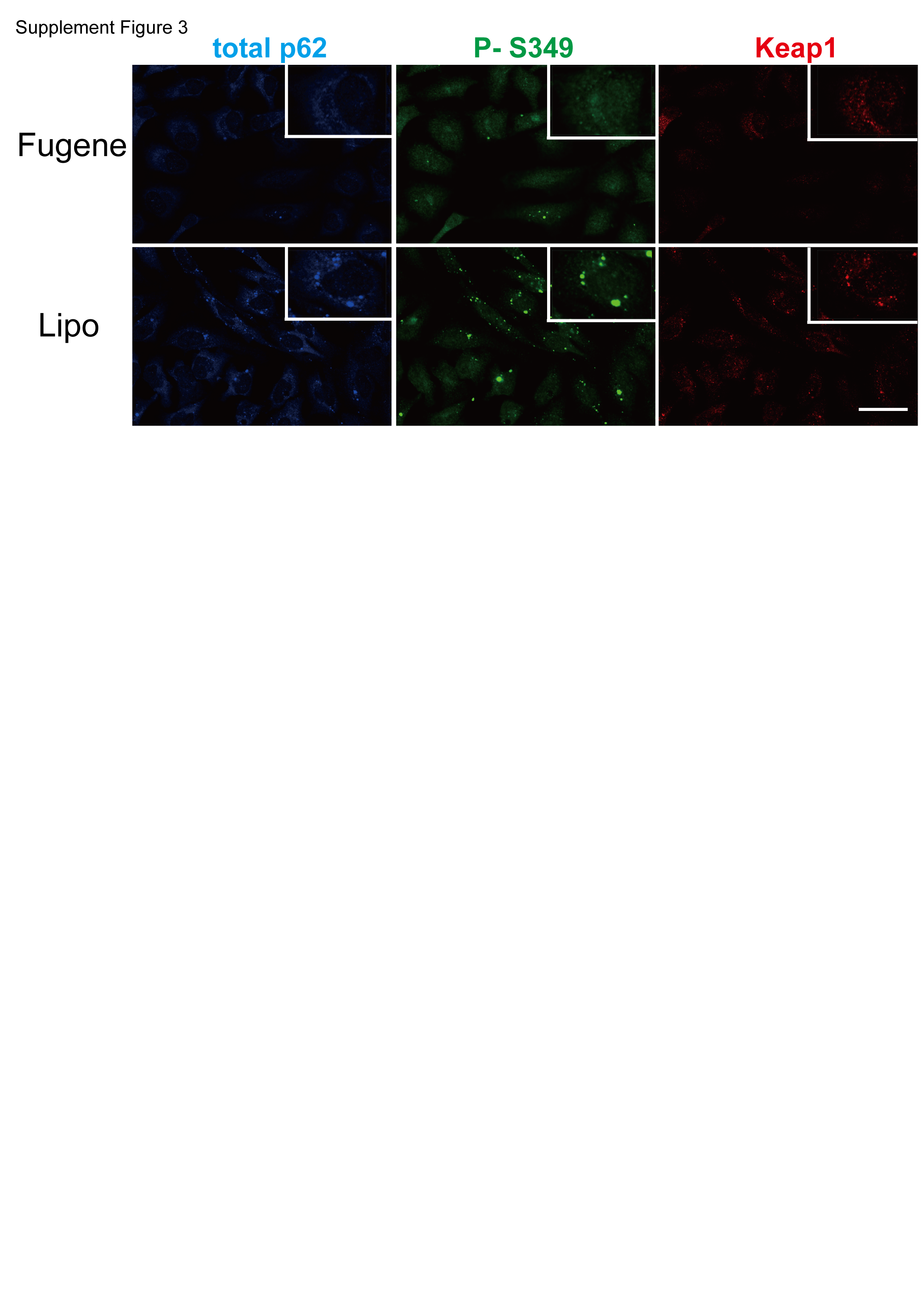

Supplement: Supplementary file 3 — Additional file 3: Figure S3: The effect of transient transfection on p62 phosphorylation. HeLa cells were treated with transfection reagent alone, Fugene 6 or Lipofectamine 2000 (Lipo) for 24 h. Confocal immunofluorescence analysis shows that total p62 (blue) forms cytoplasmic bodies, and P-S349 signals (green) are detected in these inclusions. Endogenous Keap1 (red) is weakly detected in the cytoplasm. The Keap1 signal is enhanced in cells transfected with Lipo and is partially colocalised in p62 inclusions. The islet panels indicate higher magnification. Scale bar = 20 μm. (TIFF 3 MB) [file 40478_2014_124_MOESM3_ESM.tiff]
